# Novel Genes Involved in Resistance to Both Ultraviolet Radiation and Perchlorate From the Metagenomes of Hypersaline Environments

**DOI:** 10.3389/fmicb.2020.00453

**Published:** 2020-03-26

**Authors:** María Lamprecht-Grandío, Marta Cortesão, Salvador Mirete, Macarena Benguigui de la Cámara, Carolina G. de Figueras, Danilo Pérez-Pantoja, Joseph John White, María Eugenia Farías, Ramon Rosselló-Móra, José Eduardo González-Pastor

**Affiliations:** ^1^Department of Molecular Evolution, Centro de Astrobiología (CSIC-INTA), Madrid, Spain; ^2^Programa Institucional de Fomento a la Investigación, Desarrollo e Innovación, Universidad Tecnológica Metropolitana, Santiago, Chile; ^3^Laboratorio de Investigaciones Microbiológicas de Lagunas Andinas (LIMLA), Planta Piloto de Procesos Industriales y Microbiológicos (PROIMI), Centro Científico Tecnológico, Consejo Nacional de Investigaciones Científicas y Técnicas, San Miguel de Tucumán, Argentina; ^4^Marine Microbiology Group, Department of Ecology and Marine Resources, Mediterranean Institute for Advanced Studies (IMEDEA, CSIC-UIB), Esporles, Spain

**Keywords:** UV resistance, perchlorate resistance, 4-nitroquinoline 1-oxide, DNA repair, functional metagenomics, hypersaline environments, Andean highlands

## Abstract

Microorganisms that thrive in hypersaline environments on the surface of our planet are exposed to the harmful effects of ultraviolet radiation. Therefore, for their protection, they have sunscreen pigments and highly efficient DNA repair and protection systems. The present study aimed to identify new genes involved in UV radiation resistance from these microorganisms, many of which cannot be cultured in the laboratory. Thus, a functional metagenomic approach was used and for this, small-insert libraries were constructed with DNA isolated from microorganisms of high-altitude Andean hypersaline lakes in Argentina (Diamante and Ojo Seco lakes, 4,589 and 3,200 m, respectively) and from the Es Trenc solar saltern in Spain. The libraries were hosted in a UV radiation-sensitive strain of *Escherichia coli* (*recA* mutant) and they were exposed to UVB. The resistant colonies were analyzed and as a result, four clones were identified with environmental DNA fragments containing five genes that conferred resistance to UV radiation in *E. coli*. One gene encoded a RecA-like protein, complementing the mutation in *recA* that makes the *E. coli* host strain more sensitive to UV radiation. Two other genes from the same DNA fragment encoded a TATA-box binding protein and an unknown protein, both responsible for UV resistance. Interestingly, two other genes from different and remote environments, the Ojo Seco Andean lake and the Es Trenc saltern, encoded two hypothetical proteins that can be considered homologous based on their significant amino acid similarity (49%). All of these genes also conferred resistance to 4-nitroquinoline 1-oxide (4-NQO), a compound that mimics the effect of UV radiation on DNA, and also to perchlorate, a powerful oxidant that can induce DNA damage. Furthermore, the hypothetical protein from the Es Trenc salterns was localized as discrete foci possibly associated with damaged sites in the DNA in cells treated with 4-NQO, so it could be involved in the repair of damaged DNA. In summary, novel genes involved in resistance to UV radiation, 4-NQO and perchlorate have been identified in this work and two of them encoding hypothetical proteins that could be involved in DNA damage repair activities not previously described.

## Introduction

Electromagnetic energy in the form of ultraviolet (UV) radiation is considered an extreme parameter (Rothschild and Mancinelli, 2001) and can be classified into three types: ultraviolet-A (UVA) (315–400 nm), ultraviolet-B (UVB) (280–315 nm) and ultraviolet-C (UVC) (100–280 nm), as defined by the International Commission on Illumination (Sliney, 2007). On the Earth, most of the UVC radiation is attenuated by the ozone layer, but UVA and UVB can reach the surface. Exposure to short wavelengths of ultraviolet radiation UVA and UVB causes direct and indirect damage to cells, particularly in DNA and photosystems (Sinha and Häder, 2002; Gao and Garcia-Pichel, 2011; Cadet et al., 2014).

High UV radiation exposure is believed to have been present on the early Archaean Earth, posing a challenge to life’s protection and cell repair processes (Cockell, 1998) and is currently prevailing in space environments such as the surfaces of spacecrafts and planetary bodies (Mancinelli and Klovstad, 2000). Nevertheless, microorganisms such as the Gram-positive bacteria *Bacillus subtilis* and *Deinococcus radiodurans*, and others such as members of the planctomycetes have been found thriving under this extreme condition (Battista, 1997; Nicholson et al., 2000). This has triggered scientists to question how life has evolved to resist UV-radiation (Cockell, 2000). To address this issue, several bacteria have been isolated from extreme environments impacted by high doses of UV radiation on their surfaces, including high-latitude (Quesada and Vincent, 1997) and high-altitude sites (Zenoff et al., 2006; Albarracín et al., 2012, 2015, 2016; Rasuk et al., 2017).

There is a wide array of different strategies to overcome the challenge of UV radiation. A first line of defense is provided by secondary metabolites in the form of pigments such us scytonemin, melanins and mycosporines, which can act as UV sunscreen compounds (Gao and Garcia-Pichel, 2011). If these barriers fail, a set of repair processes is used to deal with cellular damage. In fact, there is a sequence of effects triggered by UV irradiation on bacterial cells that mainly affect DNA, such as reactive oxygen species (ROS) formation and mutagenesis. It has been proposed that osmoprotectants, compatible solutes, such as glycine betaine and inositol, and carotenoid pigments could act as molecules that protect against oxidative stress (Sandmann, 2015; Pérez et al., 2017). UV radiation is among the leading contributors for most of the mutagenesis due to a process of DNA translesion synthesis (TLS) in which a polymerase or replicative assembly encounters a non-coding or miscoding lesion, inserts an incorrect nucleotide opposite the lesion and then continues elongation. UV can also originate cyclobutane pyrimidine dimers (CPDs), pyrimidine (6-4) pyrimidone photoproducts (6-4PPs) and their Dewar isomers, apurinic/apyrimidinic sites (AP sites), single-stranded breaks (SSBs) and double-stranded breaks (DSBs) (Rastogi et al., 2010). Consequently, there are several tolerance and damage-control mechanisms that allow organisms to cope with UV radiation. Efficient CPD diminution after UVB damage using photolyases has been well established in isolates from high altitude Andean environments (Albarracín et al., 2012, 2014; Kurth et al., 2015). In addition, the presence of photoactive molecules, with homology to photolyases/cryptochromes members (CPF) was characterized by phylogenetic analyses, to prove the presence of functional domains and key residues in the novel UV resistance proteins (Portero et al., 2019).

There are several different molecular processes involved in DNA damage repair such as nucleotide excision repair (NER), mismatch repair (MMR), homologous recombination (HR), non-ending homologous recombination (NEHJ), photoreactivation that acts at a lesion-directed level, base excision repair (BER) and the SOS response involved in general DNA damage repair (Friedberg et al., 2005). In addition, high UV radiation can trigger the SOS response, which is activated when there is severe DNA damage preventing DNA replication and repair from proceeding effectively. Both the LexA transcriptional repressor and the RecA recombinase regulate this system (Wertman and Mount, 1985). Thus, SSBs produced by UV radiation are recognized by the protein RecA which also catalyzes the cleavage of LexA, diminishing its levels. The cleaved LexA de-represses the regulon involved in the SOS response and allows expression of SOS responding genes. Once the DNA damage has been repaired, the RecA activity ceases, and LexA can regain the control of the SOS genes (Krishna et al., 2007; Rastogi et al., 2010). The SOS regulon comprises more than 40 genes, including those encoding proteins UmuD and UmuC, RecA, and LexA. Also forming part of this regulon are the genes encoding UvrA, B, C: a group of nucleotide excision repair (NER) proteins that locate and excise damaged regions from the DNA (Aksenov, 1999; Janion, 2008; Shah and He, 2015).

It is well known that a high proportion of the microorganisms present in a particular environment cannot be cultured under standard laboratory conditions (Giovannoni et al., 1990; Ward et al., 1990; Amann et al., 1995). As a consequence, the identification of mechanisms of adaptation to UV radiation based only on cultured microorganisms may overlook novel functions of resistance. To detect novel genes from the uncultured fraction, culture independent approaches such as functional metagenomics are employed. This molecular approach consists of the extraction of environmental DNA and the subsequent construction of gene libraries (González-Pastor and Mirete, 2010; Lam et al., 2015). The resulting clones from these libraries can then be screened for several cellular activities. This approach allows the identification of novel genes because their sequences are not derived from known nucleotide sequences and, furthermore, the genes identified during the screening are functional (Simon and Daniel, 2009; López-Pérez and Mirete, 2014; Mirete et al., 2016). In recent years, the identification of novel genes involved in adaptation to other extreme conditions present in hypersaline, acidic, cold and hot environments, has been achieved through functional metagenomics (Mirete et al., 2016). Despite the recent progress made, to our knowledge this culture independent approach has not been used to retrieve novel UV-radiation resistant genes from microorganisms of hypersaline environments. This strategy was chosen in this study to search for UV-radiation resistance genes derived from three aquatic environments highly exposed to radiation: two lakes from the Andean highlands (Argentina) and the Es Trenc solar saltern in Mallorca (Spain).

## Materials and Methods

### Bacterial Strains, Media and Culture Conditions

*Escherichia coli* DH10B strain (Invitrogen) was routinely grown in Luria-Bertani (LB) medium (Laboratorios Conda) at 37°C (Sambrook and Russell, 2001) and it was used as a host to construct the metagenomic libraries. In all experiments, the growth media for transformed *E. coli* strains was supplemented with 100 μg mL^–1^ ampicillin (Ap) to maintain the pBluescript II SK (+) plasmid. When required, the working concentration of 5-bromo-4-chloro-3-indolyl-β-galactopyranoside (*X*-Gal) was 98 μM and of isopropyl β-D-1-thiogalactopyranoside (IPTG) was 100 mM.

### Isolation of Metagenomic DNA From Brines

High-quality metagenomic DNA was obtained from brines from the Es Trenc salterns (Mallorca, Spain) and two different saline lakes from Antofagasta (Argentina): Diamante Lake in the Galán Volcano (coordinates: 26°00′ 51.04′′S, 67°01′46.42′′O; 4,589 m above sea level) and Ojo Seco Lake from Antofalla salt flat (coordinates: 25°34′06′′S, 67°35°53′′W; 3,200 m above sea level). The water from these environments is characterized by high salinity conditions and high radiation. Brines from Es Trenc solar saltern has 38.53% salinity and samples were collected in august 2012. Diamante Lake has 26% salinity, extreme alkalinity (pH 11) and high concentrations of arsenic (230 mg L^–1^) (Rascovan et al., 2016) and Ojo Seco Lake has 27% salinity and pH 8.5 (samples collected by LIMLA-PROIMI-CONICET). The samples were filtered through 0.22 μm filter (Millipore) and the filter was stored at −80°C until the DNA extraction. The metagenomic DNA was directly extracted using BIO101 FastDNA spin kit (Qbiogene) and the FastPrep device following to the manufacturer’s recommendations with no further treatment.

### Construction of Metagenomic Libraries

The construction of metagenomic libraries and their amplification was performed as previously reported (Mirete et al., 2007; González-Pastor and Mirete, 2010). Briefly, the metagenomic DNA was partially digested using *Sau*3AI. DNA fragments ranged from 1 to 8 kb were collected directly from a 0.8% low melting point agarose gel using the QIAquick Gel Extraction kit (QIAGEN) and ligated to the dephosphorylated and *Bam*HI digested pBluescript II SK (+) vector. The purified DNA (100 ng) was mixed with the vector in a molar ratio 1:1. Ligations were incubated overnight at 16°C using T4 DNA ligase (Roche) and were used to transform *E. coli* DH10B competent cells (Invitrogen) by electroporation using a Micropulser (Bio-Rad) according to the manufacturer’s instructions. Transformed cells were selected on solid LB medium supplemented with Ap, *X*-gal, and IPTG. The ratio of cells with recombinant plasmids in the library was determined by counting white (recombinant) versus blue (non-recombinant) colonies. To determine the average insert size of the library, the plasmids of 20 random recombinant clones were isolated and digested using *Xba*I and *Xho*I restriction enzymes (Roche). To amplify the libraries, they were grown on LB agar plates containing Ap (approximately 1.3 × 10^4^ cells per plate) and incubated for 24 h at 37°C. Cells from each plate were mixed with 3.5 ml LB plus 10% glycerol (w/v), pooled in a flask and stored at −80°C.

### Screening the Library for UV Resistant Clones

Independent cultures of approximately 10^8^ cells from each amplified library were directly plated on solid LB-Ap medium and irradiated at room temperature with a germicidal lamp (312 nm) operating at 2.26 W m^–2^ for 120 s, a lethal dose of radiation for DH10B pBluescript II SK (+) cells. After the UV exposure, plates were incubated for 16 h at 37°C and colonies from survivors were considered putative UV resistant clones. Surviving colonies were pooled together, the plasmids were isolated from the pool and used to transform DH10B cells. The new retransformed clones were selected on solid LB Ap medium and 150 clones were tested individually to determine UV resistance by a drop assay.

### UV Resistance Test by Drop Assay

*Escherichia coli* DH10B cells containing recombinant plasmids were grown overnight in LB-Ap and aeration at 30°C. After that, the optical density at 600 nm (OD_600_) of each culture was adjusted at 1.0 and serial dilutions were performed. Finally, a 10 μl drop of each dilution was inoculated in solid LB-Ap medium and exposed to UVB radiation (312 nm) at 2.26 W m^–2^ for 80 s or to UVC radiation (254 nm) at 1.56 W m^–2^ for 15 s. Plates were incubated overnight at 37°C. A control was performed without radiation exposure to verify that the cell viability of the cultures was similar. Each experiment was repeated at least three times.

### UV Survival Assay

To identify the survival percentage of the population exposed to UV radiation, overnight cultures of the recombinant clones grown in liquid LB medium were diluted to an OD_600_ of 1.0. Independent aliquots of 100 μl of the culture were exposed to UVB radiation (312 nm) at 2.26 W m^–2^ for 120 s. Serial dilutions were performed on LB, plated on solid LB-Ap medium, and cfu mL^–1^ were estimated after an overnight incubation at 37°C. Percentage of the surviving cells was calculated as the number of cfu mL^–1^ remaining after the ultraviolet exposure divided by cfu mL^–1^ at the initial time. Each experiment was repeated at least three times. The statistical analysis of the data was carried out using GraphPad Prism 7 program.

### *In silico* Analysis of UV Resistance Clones

The environmental DNA fragments cloned into pBluescript II SK (+) plasmids from four UV resistant colonies were sequenced on both strands with universal primers M13F and M13R and primers for primer walking by using the ABI PRISM dye terminator cycle-sequencing ready-reaction kit (PerkinElmer, Waltham, MA, United States) and an ABI PRISM 377 sequencer (Perkin-Elmer), according to the manufacturer’s instructions. Sequences were assembled and analyzed with the Editseq and Seqman programs from the DNA Star package. Prediction of potential open reading frames (ORFs) were conducted using ORF Finder and FGENESB (Solovyev and Salamov, 2011), which are available at the NCBI web page^1^,^2^. The bacterial code was selected, allowing ATG, CTG, GTG, and TTG as alternative start codons for translation to protein sequences. All the predicted ORFs longer than 90 bp were translated and used as queries in BLASTP and PSI-BLAST and their putative function was annotated based on their similarities to protein family domains by using Pfam (Protein families) available at the European Bioinformatics Institute (EMBL-EBI)^3^. Those translated ORFs with an E value above 0.001 in the BLASTP searches were considered as unknown proteins. For DNA-binding prediction, three different programs were used using default settings: iDNA-Prot| dis (Lin et al., 2011), DPP-PseAAC (Rahman et al., 2018), and DNABIND (Liu and Hu, 2013).

### Cloning of Candidate ORFs Involved in UV Resistance

The ORFs conferring UV resistance were identified by subcloning each individual ORF from the original metagenomic clones in pBluescript II SK (+) using specific primers (Supplementary Table S1). PCR amplification of the ORFs was carried out using the following reaction mixture: 50 ng of plasmid DNA, 500 μM of each of the four dNTPs, 2.5 U of *Pfu* Ultra DNA polymerase (Stratagene) and 100 nM of each forward and reverse primers, up to a total volume of 50 μl. The PCR amplification program used was as follows: 1 cycle of 5 min at 94°C, 30 cycles of 30 s at 94°C, 30 s at 54°C, 5 min at 72°C and finally 1 cycle of 10 min at 72°C. PCR amplification products were excised from agarose gels and purified using the QIAquick Gel Extraction Kit (Qiagen). Purified PCR products were then digested with the appropriate restriction enzyme (Roche) and ligated into pBluescript II SK (+). To incorporate their native expression sequences (promoters and ribosome binding sites), a region of approximately 100 bp located upstream of the start codon was also amplified. Some of the ORFs were truncated or the 5′ region was close to the polylinker sequence of the pBluescript II SK (+) vector, and they were subcloned in the same orientation as of the original clone. *E. coli* DH10B cells were transformed with the recombinant plasmids and the resulting strains were tested for UVB resistance by drop assay.

### 4-Nitroquinoline 1-Oxide Survival Assay

Overnight cultures of the recombinant clones grown in liquid LB medium were diluted to an OD_600_ of 1.0. Independent aliquots of 100 μl of the diluted culture were treated with 50 μM of 4-nitroquinoline 1-oxide (4-NQO) for 60 min, after that, the 4-NQO was removed by a double wash with LB medium. Serial dilutions were performed on LB, plated on solid LB-Ap, and cfu mL^–1^ were estimated after overnight incubation of the plates. Percentage of the surviving cells was calculated as the number of cfu mL^–1^ remaining after the 4-NQO treatment divided by the number of cfu mL^–1^ of untreated cells. Each experiment was repeated at least three times. The statistical analysis of the data was carried out using GraphPad Prism 7 program.

### Perchlorate Resistance Test by Drop Assay

*Escherichia coli* DH10B strains harboring the recombinant plasmids conferring UV resistance were grown overnight in liquid LB-Ap medium with aeration at 30°C. The cultures were diluted to an OD_600_ = 1.0 and 10-fold serial dilutions were performed. 10 μl of each dilution were inoculated in solid LB-Ap medium supplemented with 125 mM of sodium perchlorate (Sigma-Aldrich). Additionally, another set of dilutions in the same solid medium with sodium perchlorate was exposed to UVB radiation (312 nm) at 2.26 W m^–2^ for 80 s. A control was performed without perchlorate treatment and not exposed to radiation. Plates were incubated overnight at 37°C. Each experiment was repeated at least three times.

### Expression and Cellular Localization of the Hypothetical Proteins Responsible for UV Resistance From pML105 and pML6 by GFP Protein Fusion

To create a fusion of the hypothetical protein encoded by the *orf1* from pML105 with GFP, a fragment of 755 bp containing this ORF (excluding the last 7 nucleotides) and a 171 bp sequence upstream, was amplified by PCR using primers PML105-GFPF and PML105-GFPR (Supplementary Table S1). Concurrently, the fusion of the hypothetical protein encoded by the *orf1* of pML6 with GFP was also constructed using the same approach, amplifying a DNA fragment of 616 bp containing this ORF (excluding the last 10 nucleotides) and a 106 bp sequence upstream, using the primers PML6-GFPF and PML6-GFPR (Supplementary Table S1). Both DNA fragments were cloned at *Kpn*I site in pTU65 plasmid (Chalfie et al., 1994) in frame with the *gfp* nucleotide sequence to generate a C-terminal GFP fusion. DH10B cells were transformed with the resulting plasmid to obtain the strains DH10B pTU65-*orf1(105):gfp* and DH10B pTU65-*orf1(6):gfp*.

To express and localize the resulting GFP fusions, overnight cultures in LB medium of DH10B pTU65-*orf1(105):gfp*, DH10B pTU65-*orf1(6):gfp* and the strain DH10B Δ*rbsAR:cat_sfGFP* as a negative control (expressing a constitutive superfolder GPF, from our laboratory strain collection), were diluted to an OD_600_ of 0.05 in 10 ml of fresh LB medium (supplemented with Ap for the strains harboring pTU65 recombinant plasmids) and then grown to exponential phase at 37°C at 200 rpm. At approximately OD_600_ = 0.4, 100 mM IPTG (Sigma-Aldrich) was added to the cultures to induce the *lac* promoter in the pTU65 plasmid, upstream of the cloned ORFs, to express the GFP fusions properly. The cultures continued to incubate until they reached approximately OD_600_ = 0.8, then, 0.2 ml were taken and centrifuged 5 min at 5,000 rpm in an Eppendorf centrifuge 5415D, the recovered pellets were resuspended in 200 ml of PBS, treated with 4-NQO (50 mM) and incubated 5 min at room temperature. Subsequently, the cells were washed three times with PBS and centrifuged to remove the 4-NQO. Then, they were resuspended in 10 ml of PBS and treated for 2 min with DAPI (2 ml at 10 mg mL^–1^, to stain the DNA) and propidium iodide (2 ml, at 2 mg ml^–1^, to detect dead cells). Finally, the cells were washed with PBS, resuspended in 10 ml and visualized by a Leica CTR 6000 fluorescence microscope. The exposure time to take pictures was 1,200 ms for GFP and 300 ms for DAPI and propidium iodide. Images were acquired with a digital CCD camera; signal intensities were measured using the LAS X program (Leica).

## Results

### Screening for Genes Involved in UV Resistance

In order to identify genes that confer UV resistance to *E. coli*, metagenomic libraries of microorganisms from three aquatic environments highly exposed to radiation were explored: Ojo Seco (OS) and Diamante (D) lakes (both from the Andean highlands, Argentina), and the Es Trenc saltern in Mallorca, Spain (ET). The metagenomic library from microorganisms of the Es Trenc saltern was previously generated in our laboratory (Mirete et al., 2015), and contains approximately 236,000 recombinant clones. The average size of the DNA fragments cloned in the vector pBluescript II SK (+) used for the library was 2.9 kb, therefore the amount of cloned environmental DNA was approximately 685 Mb. The metagenomic libraries of the Ojo Seco and Diamante lakes samples were constructed for this study, and contain 285,000 and 170,000 recombinant clones, respectively. The average size of the cloned DNA fragments is 1.9 kb (OS) and 1 kb (D), ranging from 1 to 8 kb, based on the analysis of plasmids isolated from 20 recombinant clones from each library (Table 1), and an estimation of 541 (OS) and 170 Mb (D) of environmental DNA was cloned. Each library was amplified as described in Section “Materials and Methods.”

**TABLE 1:** Characteristics of the metagenomic libraries used in this study and results of the UV resistance screening.

Library	No. of clones	Avg. insert size (kb)^a^	Library size (MB)	No. of UV resistant clones^b^	No. of UV resistant clones^c^/ No. of unique UV clones^*d*^	References
PMB	236,250	2.9	685	111	29/1	Mirete et al., 2015
OS	285,000	1.9	541	13	1/1	This work
D	170,000	1.0	170	94	30/2	This work

*^*a*^Estimated from restriction digestion analysis of 20 random clones using either XbaI/XhoI enzymes. ^*b*^Number of UV resistance clones recovered after screening of the amplified library. ^*c*^Number of UV resistance clones recovered after retransformation. ^*d*^Number of UV resistance clones recovered after digestion with XbaI/XhoI enzymes and HindIII to identify the unique clones.*

**FIGURE 1:**
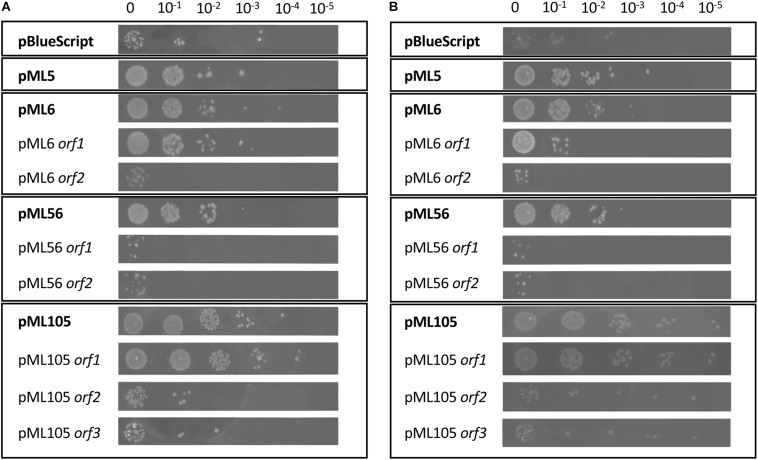
Drop assay of the four clones resistant to UV radiation and of the individual genes predicted in each clone. DH10B pBlueScript SK (+) (empty plasmid) was used as a negative control. 10 μl drops of serial dilutions of overnight cultures adjusted to an OD_600_ = 1.0 were inoculated on LB solid and expose to radiation: **(A)** UVB (312 nm), 80 s at 2.26 W m^–2^ and **(B)** UVC (254 nm), 15 s at 1.56 W m^–2^. Each experiment was performed at least three times using independent cultures.

**FIGURE 2:**
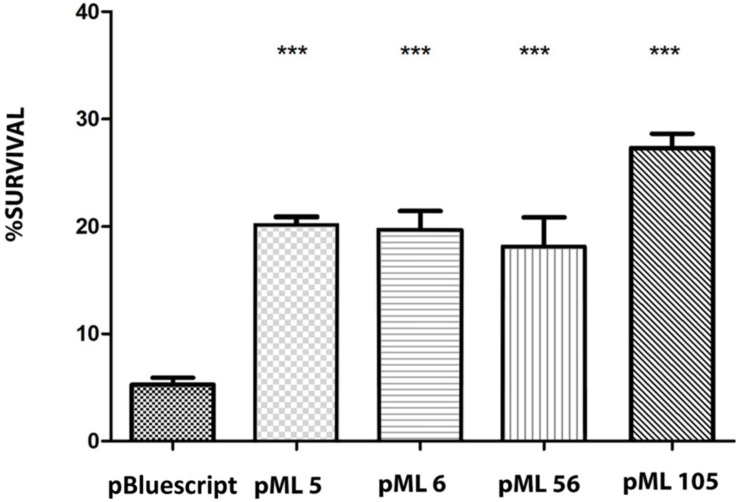
Survival percentage of the four resistant clones after exposure to 120 s of UVB (312 nm) radiation at 2.26 W m^–2^ in liquid LB medium. The significant differences are indicated by asterisks (Tukey test, ***P ≤ 0.001).

To identify UV resistant clones, aliquots from each amplified library were plated on solid LB and irradiated with a dose of UVB light lethal for the control strain *E. coli* DH10B pBluescript II SK (+). A total of 111 UVB resistant colonies were obtained from the Es Trenc (ET) library, 94 from the Diamante (D) library and 13 from the Ojo Seco (OS) library (Table 1). To exclude those clones in which UV resistance was originated by chromosomal mutations, the UVB resistant colonies from each library were pooled, the plasmids were extracted from each pool and used to transform DH10B cells. 150 colonies of each transformation were isolated on LB-Ap solid medium and tested individually for UVB radiation resistance by a drop assay, to select those containing a recombinant plasmid responsible for the resistance. The number of confirmed resistant clones from each library are listed in Table 1. The plasmids from the resistant clones were extracted and digested with *Xba*I/*Xho*I and *Hin*dIII restriction enzymes, which revealed a total of four clones with unique restriction pattern, two clones from the Diamante (D) library (pML5 and pML56), one clone from the Ojo Seco (OS) library (pML6), and one from the Es Trenc (PMB) library (pML105). These plasmids conferred resistance to UVB (2.26 W m^–2^, 80 s) and to UVC (1.56 W m^–2^, 15 s) radiation to the resulting recombinant *E. coli* clones (Figures 1A,B, and control without irradiation in Supplementary Figure S1). To quantify the level of UVB radiation resistance, the four resistant clones were assayed to determine the cell survival after exposure to UV radiation. As a result, the percent of survival cells in the case of the recombinant was increased over fourfold (between 18 and 27%) compared with the control cells as shown in Figure 2.

### Identification of Genes Conferring UV Resistance

**FIGURE 3:**
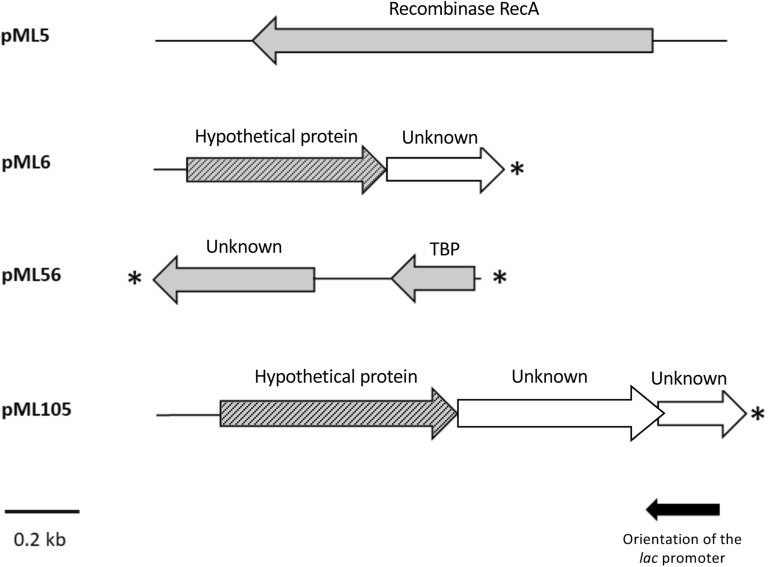
Schematic organization of the ORFs identified in plasmids pML5, pML6, pML56, and pML105. Arrows indicate the location and the transcriptional orientation of the ORFs in the different plasmids. Those ORFs implicated in the UV resistance are denoted by gray arrows. Truncated ORFs are indicated by an asterisk.

The complete double-stranded DNA sequences of each metagenomic insert in plasmids pML5, pML6, pML56, and pML105 were obtained by primer walking. The length of the inserts ranged from 0.8 to 1.6 kb and the G + C content varied from 46 to 59%, indicating a diverse phylogenetic affiliation. A total of eight open reading frames (ORFs) were predicted in the sequenced inserts, four of them truncated. Gene organization in the inserts and the similarities of the encoding proteins are summarized in Table 2 and Figure 3. Two ORFs (pML5-*orf1* and pML56-*orf2*) were similar to genes that encode proteins of known function, and other two ORFs (pML6-*orf1* and pML105-*orf1*) encoded conserved hypothetical proteins. The remaining four ORFs were not significantly similar to genes from other microorganisms.

**TABLE 2:** Description of the UV-resistant plasmids and their observed sequence similarities.

Plasmid ID	Source library	Insert length (bp)	% GC	GeneBank accession no.	ORF^*a*^	Truncated ORFs	Length (aa)^b^	Closest similar protein (microorganism; accession no.)/ Pfam domain	Identity (*E*-value)	Length (aa)	DNA binding domain prediction^d^	Putative function
pML5	D	1544	59%	MF495891	(1)	No	352	Recombinase RecA (*Tamilnaduibacter salinus*; WP 116918246.1) Pfam: PF00154 (RecA)	89% (0.0)	352	+++	Homologous recombination and DNA repair
pML6	OS	861	46%	MF495892	(1)	No	158	Hypothetical protein RHORCCE3_1666 (*Rickettsia hoogstraalii* str. RCCE3*;* KJV80064.1)	36% (5E-24)	176	++	Conserved hypothetical protein
					2	C-terminal	108	None	NA	NA	++	Unknown
pML56	D	812	49%	MF495893	(1)	C-terminal	120	None	NA	NA	None	Unknown
					(2)	N-terminal	80	TATA-box-binding protein (TBP) (*Haloferax volcanii*; WP 004042154.1) Pfam: PF00352 (TBP)	78% (8E-37)	180	None	Transcription factor
					(1)	No	189	Hypothetical protein CBD42_12610 (Gammaproteobacteria bacterium TMED182; OUW42978.1)	49% (2E-39)	162	++	Conserved hypothetical protein
pML105	PMB	1547	48%	MF495895	2	No	233	None	NA	NA	+++	Unknown
					3	C-terminal	90	None	NA	NA	+	Unknown

*^*a*^ORFs involved in UV resistance are indicated in parentheses. ^*b*^aa, amino acids. ^*c*^Those sequences with an E-value higher than 0.001 in BLASTP searches were considered to be unknown proteins. ^*d*^Each (+) sign means a positive prediction with one of the four bioinformatics programs used to predict DNA binding domains described in Section “Materials and Methods.”*

The recombinant plasmid pML5 contained a single ORF, encoding a protein highly similar (89% of identity) to the recombinase RecA from *Tamilnaduibacter salinus*, a halotolerant gammaproteobacterium (Table 2), and therefore it may be complementing the *recA* mutation of the DH10B strain and be responsible for the UV resistance observed. RecA has been described previously that operates in DNA repair by direct binding to DNA lesions (Renzette et al., 2005; Kreuzer, 2013) and, as expected, DNA-binding domains were predicted in the protein sequence encoded by the gene recovered using three different bioinformatic programs (Table 2).

**FIGURE 4:**
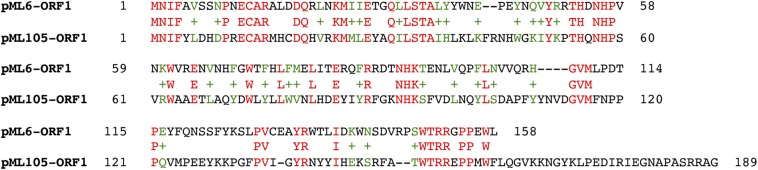
Alignment of the hypothetical proteins encoded by pML105-*orf1* and pML6-*orf1* using BLASTP.

The other recombinant plasmids contained between two and three ORFs (Table 2). To determine which ones were responsible for the UV resistance phenotype of each clone, they were individually subcloned into the vector pBluescript II SK (+), expressed in the DH10B strain and tested for their ability to resist to UV by drop assays (Figure 1). In the case of the plasmids pML6 and pML105, whose inserts contain two and three and two ORFs, respectively, only a single gene (*orf1* in both plasmids) might be responsible for the increased UV resistance of the host. pML6-*orf1* encodes a protein similar (36% identity) to a conserved hypothetical protein from the tick-associated bacterium *Rickettsia hoogstraalii* str. RCCE3, and pML105-*orf1* encodes a protein similar (49%) to a conserved hypothetical protein from the Gammaproteobacteria bacterium TMED182 (Table 2). Strikingly, when the sequence of these two hypothetical proteins were aligned using BLASTP, we found that they showed 32% of identity and a similarity of 49% (Figure 4). Although the similarity was not high, it was distributed along the sequence of the proteins, so this could indicate that both proteins are homologous with a similar function. By studying these protein sequences in more detail through three different programs for the prediction of DNA-binding domains, all consistently identified them as putative DNA-binding proteins, which could support our hypothesis that these proteins may be homologous with a similar function.

In the case of plasmid pML56 neither of the two ORFs tested separately conferred UV resistance to *E. coli*, suggesting that both ORFs were necessary to confer UV resistance. The *orf1* encoded for an unknown protein and the *orf2* encoded for an N-terminal truncated protein (80 aa) similar (78% identity) to a transcription factor, the TATA-box binding protein (TBP) from the archaea *Haloferax volcanii* (180 aa) and contains one Pfam domain PF00352 (TBP family) involved in DNA binding (Table 2).

In summary, a total of five genes conferring UV resistance to *E. coli* were retrieved from microorganisms of environments highly exposed to radiation. Two of them encoded proteins with known functions (pML5 and pML56-*orf2*), but the other three genes (from pML6, pML56-*orf1*, and pML105) encoded unknown and conserved hypothetical proteins of unknown function, which could be involved in novel mechanisms of UV resistance.

### UV Resistance Genes Also Confer Resistance to 4-NQO, a Compound That Induces DNA Damage

**FIGURE 5:**
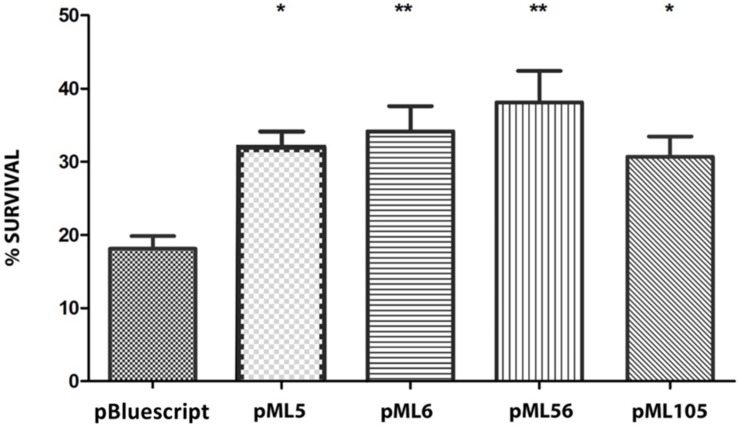
Survival percentage of the four UV-resistant clones after exposure to 60 min to 4-NQO (50 μM) in liquid LB medium. The significant differences are indicated by asterisks (Dunnett test, *P ≤ 0.05, **P ≤ 0.01, ***P ≤ 0.001).

To investigate if the mechanism of action of these genes to confer UV resistance in *E. coli* is related to protection or repair of DNA damage, we used the chemical compound 4-nitroquinoline 1-oxide (4-NQO), which mimics the effect of UV radiation on the DNA (Williams et al., 2010; Han et al., 2017). For this purpose, cultures of DH10B carrying plasmids pML5, pML6, pML56, and pML105 were tested against 4-NQO. The resistance levels conferred by the selected clones are shown in Figure 5. All the recombinant clones exhibited significantly higher survival (between 31.97 and 38.13%) than the negative control (18.1%), DH10B cells harboring an empty plasmid pSKII^+^ (Figure 5).

As described in the previous section, DNA-binding domains have been predicted by bioinformatic analysis (Table 2) in the two hypothetical proteins encoded by pML6-*orf1* and pML105-*orf1*, in the RecA protein encoded by pML5-*orf1* and in pML56-*orf2* (Pfam domain PF00352). This, together with the fact that all clones exhibited resistance to 4-NQO, suggests that the UV radiation resistance produced by the recovered environmental genes could be related to the protection or repair of DNA damage.

### The Genes Conferring UV Resistance Also Provide Perchlorate Resistance

In order to analyze whether the four clones that are resistant to UV radiation are also resistant to other stress conditions, the effect of the oxidizing agent sodium perchlorate was tested on them. In addition, the effect of the two types of stress was also tested simultaneously, since it was described that the combined effect of perchlorate with UV radiation significantly decreases the viability of bacterial samples compared to the effect of UV radiation alone (Wadsworth and Cockell, 2017).

**FIGURE 6:**
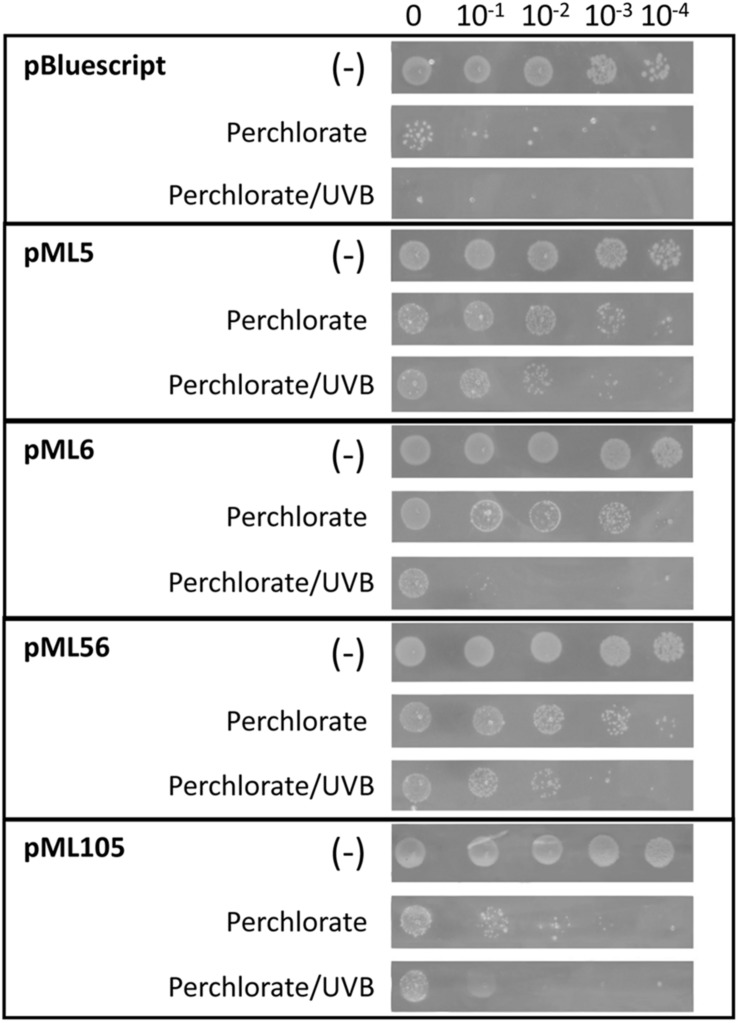
Drop assay of the four clones resistant to UV radiation to test perchlorate resistance. DH10B pBlueScript SK (+) was used as a negative control. 10 μl drops of serial dilutions of overnight cultures adjusted to an OD_600_ = 1.0 were inoculated on LB solid medium supplemented with perchlorate 125 mM. An additional test was performed combining UVB (312 nm) radiation exposure during 80 s at 2.26 W m^–2^ and perchlorate (125 mM). A negative control without irradiation (–) was performed to verify the normal growth of the clones. Each experiment was performed at least three times using independent cultures.

For this purpose, cultures of DH10B cells carrying plasmids pML5, pML6, pML56, and pML105 were tested by drop assay on solid LB medium supplemented with sodium perchlorate and also treated with the same concentration of perchlorate combined with UVB radiation. The recombinant clones pML5, pML6, and pML56 are resistant to perchlorate at least three orders of magnitude more than the control DH10B pBluescript II SK (+) and the pML105 two orders of magnitude more (Figure 6). When perchlorate and UVB radiation treatments are combined, the resistance level conferred by the recombinant clones decreases, but is still higher compared with the control, with pML5 and pML56 being three orders of magnitude higher, and pML6 and pML105 one order of magnitude higher (Figure 6).

### Cellular Localization of the Hypothetical Protein Encoded by pML105-*orf1* Is Affected by DNA Damage

The hypothetical proteins encoded by pML6-*orf1* and pML105-*orf1*, which share a similarity of 49%, could be involved in the repair or protection against DNA damage, as indicated by the experiments with 4-NQO and the presence of DNA-binding domains predicted by bioinformatics analysis. In order to explore that putative role, we decided to investigate the location of the hypothetical protein encoded by pML6-*orf1* and pML105-*orf1* in the presence of DNA damage. To this end, GFP was fused at the C-terminus to this protein. The strains expressing the fusion proteins were tested for UVB radiation resistance, to determine if they were still functional. Only the strain DH10B pTU65-*orf1(105):gfp* exhibited UV radiation resistance but not the strain DH10B pTU65-*orf1(6):gfp* (Supplementary Figure S2). Therefore, only the pML105-*orf1 GFP* fusion was functional.

**FIGURE 7:**
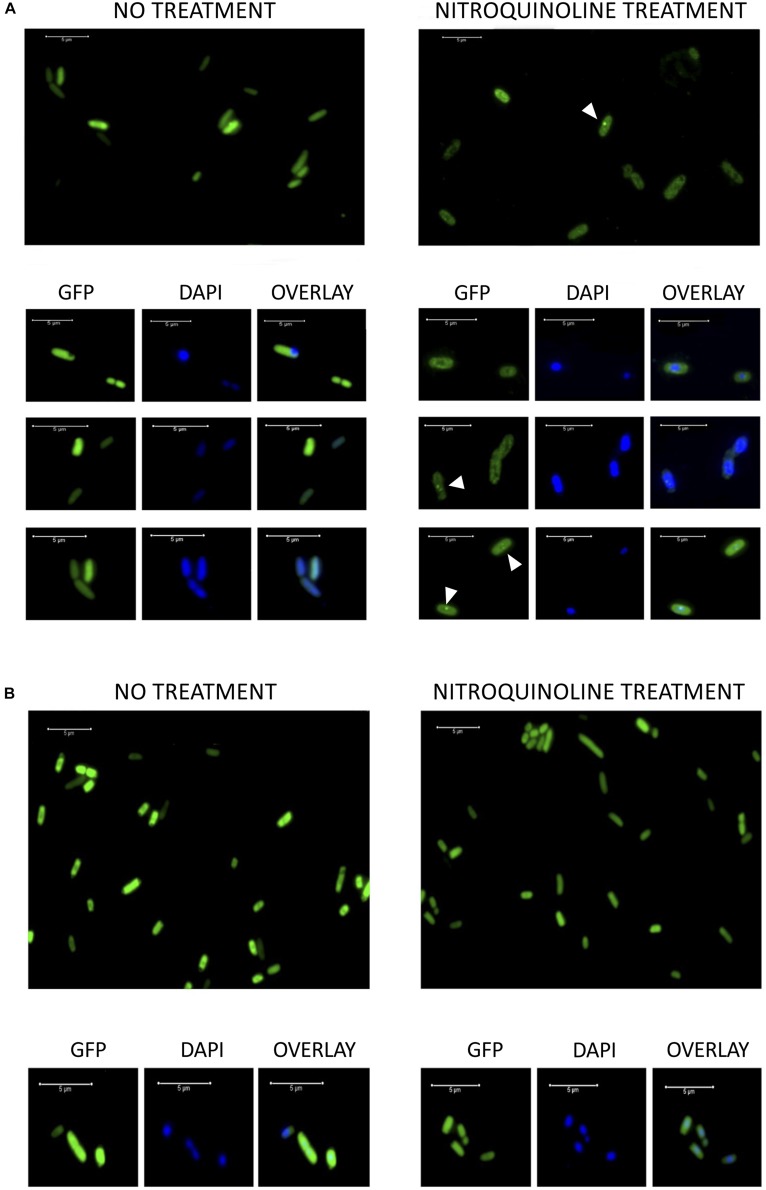
**(A)** Fluorescence microscopy images of DH10B cells expressing the hypothetical protein encoded by pML105-*orf1* gene fused to GFP [DH10B pTU65-*orf1*(105):*gfp*]. On the left, cells growing exponentially before treatment with 4-NQO. On the right, cells from the same culture treated with 4-NQO (50 μM). White arrowheads indicate bright fluorescence foci. Below, microscopy images of selected cells are observed (GFP, DAPI staining and the overlay of both images). **(B)** Fluorescence microscopy images of DH10B cells expressing GFP constitutively [DH10B Δ*rbsAR:cat_sf-gfp*]. On the left, cells growing exponentially before treatment with 4-NQO. On the right, cells from the same culture after treatment with 4-NQO (50 μM). Below, microscopy images of selected cells are observed (GFP, DAPI staining and the overlay of both images). White bars indicate 5 μm scale.

Exponentially growing DH10B pTU65-*orf1(105):gfp* and DH10B pTU65-*orf1(6):gfp* cells were observed by fluorescence microscopy before and after treatment with 4-NQO to induce DNA damage. Before treatment, it was observed that the fluorescent hypothetical protein encoded by pML105-*orf1:gfp* had a homogeneous distribution in the cytoplasm (Figure 7A). However, after 4-NQO treatment, the GFP fusion protein formed discrete foci frequently overlapping with the nucleoid as revealed by DAPI staining (Figure 7A). One possible interpretation is that the hypothetical protein recognizes and binds to the damaged sites in the DNA and possibly multimerizes, forming the observed foci. This could support the hypothesis that this protein would be involved in DNA repair. In the case of the hypothetical fluorescent protein encoded by pML6-*orf1:gfp*, no fluorescence differences were observed before and after the 4-NQO treatment as expected since the resulting GFP fusion protein was not functional. In addition, a low number of cells showed fluorescence (data not shown) compared to the construction with pML105 *orf1:gfp*.

As a control, the strain DH10B Δ*rbsAR:cat_sf-gfp*, which expresses a superfolder GFP constitutively, was used. In this case, the GFP was localized identically before and after treatment with 4-NQO, homogeneously distributed in the cytoplasm (Figure 7B). The formation of foci was not observed after the treatment, which led us to rule out that the observed location for the hypothetical protein encoded by pML105-*orf1* fused with GFP could be an artifact. On the other hand, we verified that treatment with 4-NQO did not affect cell viability in these experiments, using propidium iodide to stain dead cells (data not shown), thus it was also excluded that the presence of foci was due to cell death.

## Discussion

Previous metagenomics studies in some hypersaline environments have shown that the microbial fraction present is mainly dominated by diverse uncultivated microorganisms (Narasingarao et al., 2012; López-López et al., 2013). In this work, a functional metagenomics approach was selected to search for UV radiation resistance genes in microorganisms from hypersaline environments, since it allows access to the potential genetic diversity of both cultivated and uncultivated microorganisms. An advantage of this strategy is that functional genes can be isolated without prior knowledge of their sequences (Simon and Daniel, 2009; Mirete et al., 2016). This approach was previously used to study mechanisms of adaptation by which microbial communities cope with diverse extreme conditions, such as metals (Mirete et al., 2007; Morgante et al., 2015), acidic pH (Guazzaroni et al., 2013) and high salinity (Kapardar et al., 2010; Culligan et al., 2012, 2013; Mirete et al., 2015).

One limitation for the functional screening of metagenomic libraries is that it relies on the proper expression of heterologous proteins in *E. coli*, which sometimes cannot be achieved (Ekkers et al., 2012). Nevertheless, a total of five genes that confers UV resistance from three geographically distant environments exposed to high doses of solar radiation were successfully identified in this study. Interestingly, two of these genes showed less than 50% of amino acid identity with the closest BLASTP hit and one did not display similarity with known genes, which highlights the novelty of the encoded proteins. The lack of sequence conservation with that of other proteins previously described, supports the notion that culture-independent techniques allow access to novel genetic diversity and that through functional screenings genes can be identified that would not be discovered by other methods, such as bioinformatic analysis.

The resistance to UV radiation could be related to: (i) DNA protection or damage repair, (ii) cellular protection by pigments, or (iii) reduction of the damage produced by photo-oxidative products (Gao and Garcia-Pichel, 2011). Our results suggest the genes isolated in this study may be involved in the protection or repair of DNA damage. First, all clones showed resistance to the compound 4-NQO, which is considered a chemical analog of UV radiation because it produces DNA lesions that could be treated by the nucleotide excision repair (NER) pathway, and several studies have identified that mutants deficient in NER are both hypersensitive to UV radiation (254 nm) and to 4-NQO (Ikenaga et al., 1975). And second, DNA-binding domains were predicted in four of the five proteins involved in UV resistance.

On the other hand, all the clones exhibited resistance to perchlorate. This compound is a powerful oxidizing agent that induces bactericidal effects and can be found naturally in arid environments such as the Atacama Desert (Catling et al., 2010). Several species of hyperhalophilic archaea have been shown to be resistant to high concentrations of perchlorate (Oren et al., 2014) and, in addition, microorganisms resistant to this compound have been isolated from other types of extreme environments (Matsubara et al., 2017). However, the mechanisms and genes specifically involved in resistance to this compound are still unknown. One study has shown that perchlorate could cause DNA damage in some rat tissues due to the alteration of its structure affecting hydrogen bonding and other electrostatic interactions (Yu et al., 2019), which supports that the genes identified could be involved in the protection against DNA damage. In addition, it was described that the combination of perchlorate and UV radiation produces the generation of deleterious ROS, specifically hypochlorite and chlorite that increase cellular damage by oxidation stress (Wadsworth and Cockell, 2017). In this study, the four UV-resistant clones showed significant resistance to the combined UV and perchlorate treatment, but a decrease in resistance was observed compared to the perchlorate treatment alone, perhaps due to the high increase in ROS produced for the combined exposure.

From the genes identified in this study, the closest BLASTP hit of the protein encoded by pML5-*orf1* was a RecA from *T. salinus*, a halotolerant gammaproteobacterium isolated from a salt pan in India (Verma et al., 2015). This result was expected since the screening was performed in the *recA* mutant *E. coli* strain DH10B.RecA is a well characterized protein involved in SOS response (Sancar and Sancar, 1988) and it operates in DNA repair by direct binding to DNA lesions (Renzette et al., 2005; Kreuzer, 2013). RecA is a highly conserved protein and the gene that encodes this protein has been widely employed as a phylogenetic marker using previously known sequences derived from PCR amplification or from large data sets (Eisen, 1995; Sandler et al., 1999; Wu et al., 2011). In this study, we have recovered a complete gene that encodes a functional RecA protein that confers UV resistance of a bacterium from a natural environment without using previously known sequences. RecA was initially identified and characterized genetically in a screen for recombination deficient mutants of *E. coli* and was found to have multiple roles in recombination and repair (Lusetti and Cox, 2002; Patel et al., 2010). This protein has four known functions in the *E. coli* cell: (i) it catalyzes the DNA strand exchange reaction in the context of recombinational DNA repair, (ii) the induction of the SOS response to UV irradiation by promotion of the autocatalytic cleavage of the LexA repressor, (iii) activation of UmuD′ (component of the DNA polymerase V involved in DNA repair) by mediating autocatalytic cleavage of UmuD, and (iv) direct participation in SOS mutagenesis by activation of DNA polymerase V. It has been shown that in SOS-uninduced cells, RecA is present at less than 10,000 monomers per cell, but upon SOS induction the level of RecA can increase to over 70,000 monomers per cell (Krishna et al., 2007). The bacterial RecA protein is a DNA-dependent adenosine triphosphatase (ATPase) and in *E. coli* hydrolyses adenosine triphosphate (ATP) at a rate of 20–30 molecules per minute, depending on the nature of the bound DNA. RecA binds to DNA as a nucleoprotein filament that forms most rapidly onto single-stranded DNA (Patel et al., 2010). The recovery of a gene encoding RecA from a metagenome, confirms the methodology used in this study as a valid approach to find genes involved in resistance to UV radiation.

An interesting finding derived from this study was the identification of two hypothetical proteins encoded by pML6-*orf1* and pML105-*orf1*, which confer UV resistance and which can be considered homologous based on their significant similarity (49%) when analyzed with BLASTP. It is interesting to note that these genes come from two distant geographic sites: pML6 from the Ojo Seco Andean Lake in Argentina and pML105 from the Es Trenc saltern, in Mallorca, Spain. In relation to the mechanism of resistance to UV radiation of these proteins, several lines of evidences support their involvement in DNA damage repair: (i) resistance to 4-NQO and perchlorate, (ii) prediction of DNA binding domains, and (iii) the cellular localization of the protein encoded by pML105-*orf1*, fused with GFP, changes after incubation with 4-NQO, and forms discrete foci possibly associated with damaged sites in the DNA. A similar pattern of localization, foci formation after induction of DNA damage, has been observed in other proteins involved in DNA damage repair (Renzette et al., 2005; Mascarenhas et al., 2006; Sanchez et al., 2006).

The environmental DNA fragment of clone pML56 was found to be involved also in resistance to 4-NQO and perchlorate, which suggests that it would also be related to DNA protection or damage repair. Simultaneous expression of the two putative genes that were identified in this fragment would be responsible for that resistance: the *orf1* encoding an unknown protein and the *orf2* encoding a TATA-box binding protein (TBP) from the archaea *Haloferax volcanii*. The TBP is a general transcription factor that binds specifically to a DNA sequence called the TATA box, which is upstream of the transcription start site in archaea and eukaryotes (Kramm et al., 2019). The TBP proteins of *Haloferax* and other archaea contain two copies of the Pfam domain PF00352 involved in the binding to the TATA box. Interestingly, one copy of this domain is found in the truncated protein encoded by *orf2*. Therefore, it could be functional and bind to TATA sequences in the *E. coli* genome, somehow protecting the DNA from UV radiation, although only when expressed simultaneously with the unknown protein encoded by *orf1*. In fact, it has been proposed that the primary ancestor of TBP could have functioned as a dimeric DNA binding protein before gene duplication and fusion would have produced the current structure of this family of proteins (Nikolov et al., 1992).

To our knowledge, functional metagenomics has never been applied to search for genes that confer resistance to UV radiation in microorganisms. Thus, this represents the first approach in which this methodology has been successfully applied in the identification of novel UV-resistance genes, such as those encoding two hypothetical proteins that could be involved in DNA damage repair activities not previously described. Furthermore, this is the first report of bacterial genes that confer resistance to the toxic perchlorate compound, which will contribute to a better understanding of the mode of action and mechanisms of resistance to this compound in microorganisms. On the other hand, it cannot be excluded that some of the genes identified may be related to the defense against oxidative stress, which can be induced by UV, 4-NQO and perchlorate. Nevertheless, further *in silico and in vitro* characterization of the proteins encoded by the identified genes will be necessary to find out the molecular mechanisms behind UV radiation and perchlorate resistance.

## Data Availability Statement

The datasets generated for this study can be found in the GenBank. https://www.ncbi.nlm.nih.gov/nuccore/MF495895, https://www.ncbi.nlm.nih.gov/nuccore/MF495893, https://www. ncbi.nlm.nih.gov/nuccore/MF495892, and https://www.ncbi.nlm.nih.gov/nuccore/MF495891.

## Author Contributions

ML-G and JG-P designed the experiments. ML-G, SM, and MF constructed the metagenomic libraries. ML-G performed the screening, analysis of clones, and microscopy. MC, MB, and CF collaborated in the analysis of the resistant clones. DP-P performed the bioinformatic analysis. JW constructed the *E. coli* strain DH10B Δ*rbsAR:cat_sfGFP*. MF, RR-M, and JG-P collected the environmental samples. ML-G, SM, and JG-P wrote the manuscript.

## Conflict of Interest

The authors declare that the research was conducted in the absence of any commercial or financial relationships that could be construed as a potential conflict of interest.

## Footnotes

1 https://www.ncbi.nlm.nih.gov/orffinder/

2 www.softberry.com

3 http://pfam.xfam.org/
