# Supplementary figures and images for: Novel Genes Involved in Resistance to Both Ultraviolet Radiation and Perchlorate From the Metagenomes of Hypersaline Environments
Source: Front Microbiol. 2020 Mar 26;11:453. doi: 10.3389/fmicb.2020.00453 (PMC7135895; doi:10.3389/fmicb.2020.00453)

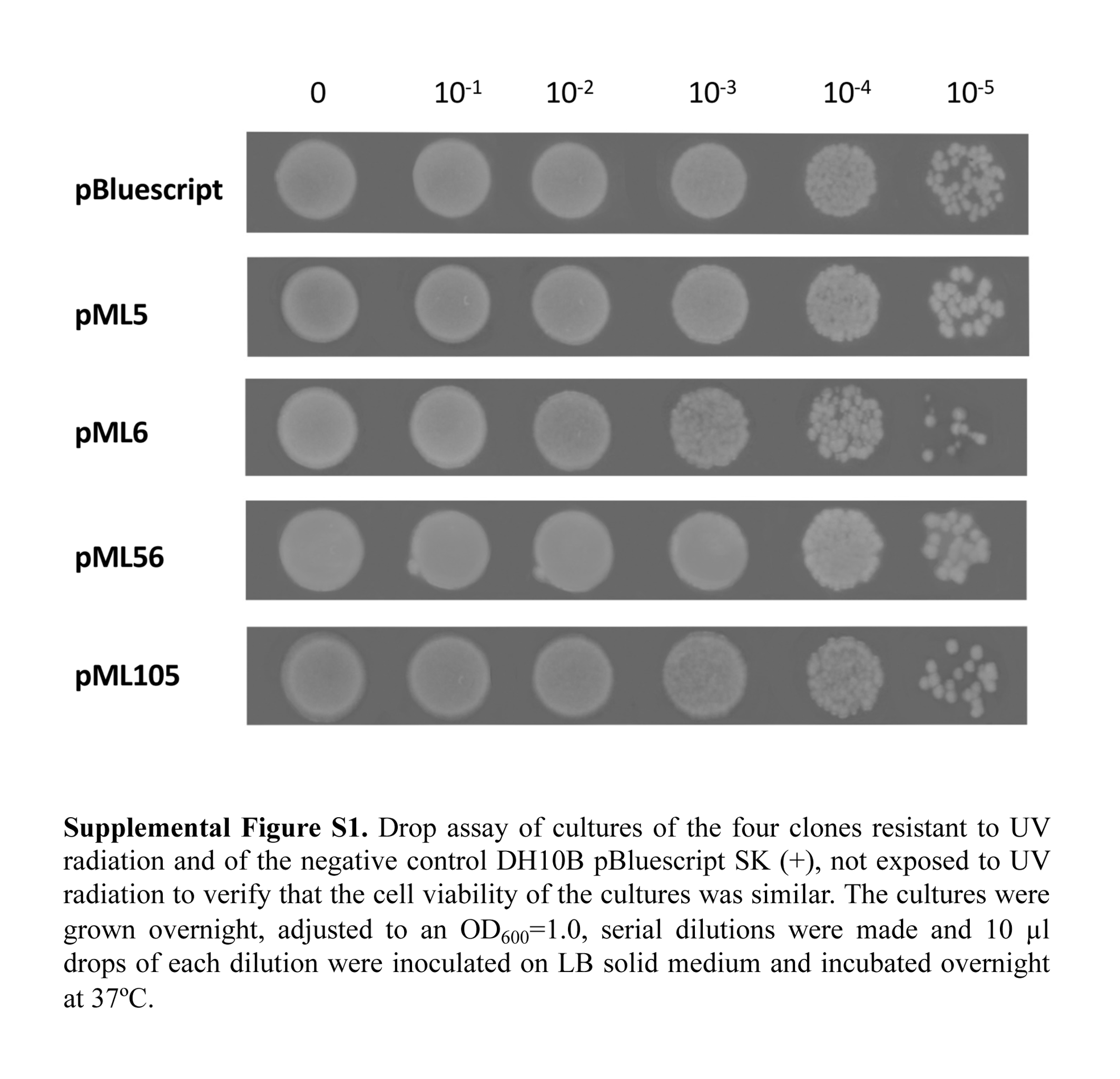

Supplement: Supplementary file 1 [file Image_1.TIF]

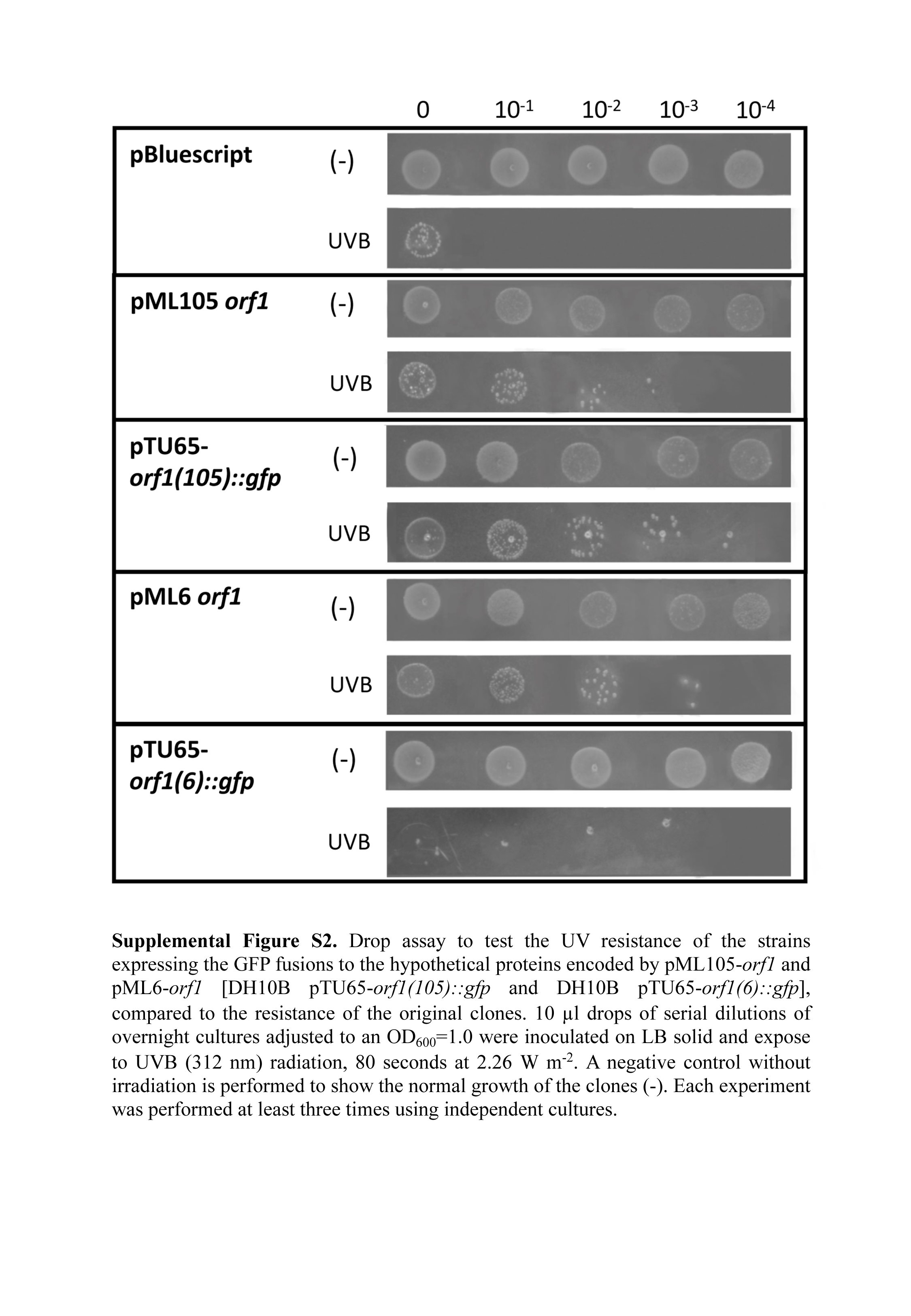

Supplement: Supplementary file 2 [file Image_2.TIF]
