# Supplementary material for: Novel Genes Involved in Resistance to Both Ultraviolet Radiation and Perchlorate From the Metagenomes of Hypersaline Environments
Source: Front Microbiol. 2020 Mar 26;11:453. doi: 10.3389/fmicb.2020.00453 (PMC7135895; doi:10.3389/fmicb.2020.00453)
Supplement: Supplementary file 3 [file Table_1.pdf]

Supplemental Table S1. Primers used in this work.

Primer name	Sequence (5´-3´)	Restriction enzyme	PCR length product (bp)
pML6-ORF1F	CAGGTCTCGAGGTATCGATAAGCTTGATATCG	XhoI	836
pML6-ORF1R	TTAGTCTAGACAATCACTCGGAAACTTAGG	XbaI	
pML6-ORF2F	TGACGGATCCAAACCACTTTGGTTGGACG	BamH1	641
pML6-ORF2R	TTAGCTCGAGCCCATTACCTCACTAAAGG	XhoI	
pML56-ORF1F	TTAGCTGCAGGGTATCGATAAGCTTGATATC	Pst1	606
pML56-ORF1R	TGACCTCGAGAGATTTGTGACTTTGTATCTCG	XhoI	
pML56-ORF2F	TTAGCTGCAGGGTCAGGAAGCACTCTTCAG	Pst1	425
pML56-ORF2R	TTAGCTCGAGCCAAGCGCGCAATTAACCCT	XhoI	
pML105-ORF1F	ATTCCGCTCGAGTCGAGGTCGACGGTATCG	XhoI	847
pML105-ORF1R	ATTCCGTCTAGAATCGAAGTAAAACCTCGCGC	XbaI	
pML105-ORF2F	ATTCCGAAGCTTGAATCAGTATCTCTCTGATGC	HinDIII	735
pML105-ORF2R	ATTCCGGAATTCCGAGTTGTTTGTGTTCCTCG	EcoRI	
PML105-GFPF	ATTCCGGGTACCGTATCGATAAGCTTGATATCG	KpnI	755
PML105-GFPR	ATTCCGGGTACCGCGCGTCTGGATGCTGGA	KpnI	
PML6-GFPF	ATTCCGGGTACCGTATCGATAAGCTTGATATCG	KpnI	616
PML6-GFPR	ATTCCGGGTACCTCAGGCGGTCCACGTCG	KpnI	
